# Prevalence and Mortality Rates of Acute Kidney Injury among Critically Ill Patients: A Retrospective Study

**DOI:** 10.1155/2023/9966760

**Published:** 2023-11-16

**Authors:** Randa I. Farah, Othman A. Alfuqaha, Ali R. Younes, Hasan A. Mahmoud, Alhareth M. Al-Jboor, Mohammad M. Karajeh, Mohammad Z. Al-Masadeh, Omar I. Murad, Nathir Obeidat

**Affiliations:** ^1^Nephrology Division, Internal Medicine Department, School of Medicine, The University of Jordan, Amman 11942, Jordan; ^2^Counseling and Mental Health Department, Faculty of Educational Sciences, The World Islamic Sciences & Education University W.I.S.E, Amman 11947, Jordan; ^3^School of Medicine, The University of Jordan, Amman 11942, Jordan; ^4^Pulmonary Critical Care Division, Internal Medicine Department, School of Medicine, The University of Jordan, Amman 11942, Jordan

## Abstract

Acute kidney injury (AKI) poses a significant challenge in critically ill patients. To determine the prevalence, risk factors, and mortality rate of AKI among nonsurgical critically ill patients in Jordan University Hospital, we conducted a retrospective study using a consecutive sampling method, including 457 nonsurgical critically ill patients admitted to the medical intensive care unit (MICU) from January to June 2021. The mean age was 63.8 ± 18 years, with 196 (42.8%) developing AKI during their stay in the MICU. Among AKI nonsurgical patients, pulmonary diseases (*n* = 52; 34.5%) emerged as the primary cause for admission, exhibiting the highest prevalence, followed by sepsis (*n* = 40; 20.4%). Furthermore, we found that older age (adjusted OR (AOR): 1.04; 95% confidence interval (CI): 1.04–1.06; *p* = 0.003), preadmission use of diuretics (AOR: 2.12; 95% CI: 1.06–4.25; *p* = 0.03), use of ventilators (2.19; 95% CI: 1.12–2.29; *p* = 0.02), and vasopressor use during MICU stay (AOR: 4.25; 95% CI: 2.1308.47; *p* = 0.001) were observed to have higher mortality rates. Prior utilization of statins before admission exhibited a significant association with reduced mortality rate (AOR: 0.42; 95% CI: 0.2–0.85; *p* = 0.02). Finally, AKI was associated with a higher mortality rate during MICU stay (AOR: 2.44; 95% CI: 1.07–5.56; *p* = 0.03). The prevalence of AKI among nonsurgical patients during MICU stay is higher than what has been reported previously in the literature, which highlights the nuanced importance of identifying more factors contributing to AKI in developing countries, and hence providing preventive measures and adhering to global strategies are recommended.

## 1. Introduction

Acute kidney injury (AKI) poses a significant challenge in critically ill patients, leading to substantial morbidity and mortality rates while also heightening the risk of both short-term and long-term complications [1]. The prevalence of AKI reported in critically ill patients varies substantially [2]. The absence of a consensus on AKI has led to a broad spectrum of estimated prevalence within the intensive care unit (ICU), spanning from 1% to 70%. These variations in prevalence are contingent upon the specific criteria employed for AKI diagnosis including creatinine serum level and urine output [2]. In Canada, the prevalence of AKI among critically ill patients has been reported to be 64.2% [3]. Many developing countries may have restricted resources for conducting research on AKI. As a result, AKI may be underdiagnosed or not well tracked in developing countries, making it difficult to estimate its prevalence and outcomes [4].

Numerous risk factors have been identified for the onset of AKI, encompassing conditions like heart and pulmonary diseases, liver failure, sepsis, advanced age, preexisting renal disease, and various medications including angiotensin-converting enzyme inhibitors (ACEIs), vasopressors, aminoglycosides, and nonsteroidal anti-inflammatory drugs (NSAIDs) [5]. Significant differences exist in the prevalence, risk factors, and mortality related to AKI among different countries, as well as among different areas with varying socioeconomic statuses and different hospital facilities [6]. Regarding this, length of stay in ICU [7], older age above 60 years old [8], female gender [9], and other chronic diseases [10] were found to be associated with AKI. They were poorly investigated in Arab countries, particularly in Jordan.

People in developing countries (i.e., Jordan) may develop more AKI than people in developed countries because of several factors such as medication nonadherence [11], higher poverty rates [12], and higher anxiety levels [13]. Due to the scarcity of literature on AKI among nonsurgical critically ill patients in our region, it is noteworthy that only one study conducted in Jordan has investigated the prevalence of AKI in the northern district of the country. Furthermore, it is important to consider that the data collection for that study took place some time ago and may not accurately reflect the current state of AKI development [14]. Therefore, the aim of our study is to determine the prevalence of AKI in nonsurgical critically ill patients admitted to the medical intensive care unit (MICU) at Jordan University Hospital (JUH). Additionally, we aim to assess the related risk factors and mortality rate in this patient population.

## 2. Materials and Methods

### 2.1. Study Design

To accomplish the aim of our study, we employed a retrospective study design involving nonsurgical critically ill patients.

### 2.2. Ethical Approval

The JUH institutional review board (no. 10/2022/5678) approved this study. All procedures conducted in this study adhered to the ethical standards set by the institutional and/or national research committee and followed the principles outlined in the World Medical Association Declaration of Helsinki. Due to the retrospective nature of the study, the requirement for informed consent from patients or their families was waived. We kept all data in a secure place to ensure the anonymity of their identity.

### 2.3. Setting

We conveniently selected the JUH, which is the largest hospital in Amman, Jordan, to represent the study population, as it is considered the largest tertiary hospital and has the largest number of ICU beds in Jordan. It provides medical services to population across the central, eastern, and southern regions of the country. The inclusion criteria for patient selection encompassed patients aged 18 years and older who were admitted to ICUs with medical conditions, including pulmonary, gastroenterology, and neurological diseases. Additionally, patients with infections and cardiovascular conditions not requiring surgical intervention were also considered eligible for inclusion. The study excluded patients who were under 18 years of age and had surgical conditions due to the inherent risk of AKI associated with surgery. Furthermore, patients with AKI at ICU admission, end-stage renal disease requiring dialysis, recent myocardial infarction, or those necessitating cardiac interventions were also excluded to maintain the scope and focus of the study.

### 2.4. Participants

The study included the entirety of nonsurgical critically ill patients who were admitted to MICUs during the period from January to June 2021. The selected hospital has several ICUs such as neuro, surgical, and medical. However, we only considered the MICU as it is the biggest unit in Jordan with a capacity of 36 beds [15].

**Figure 1:**
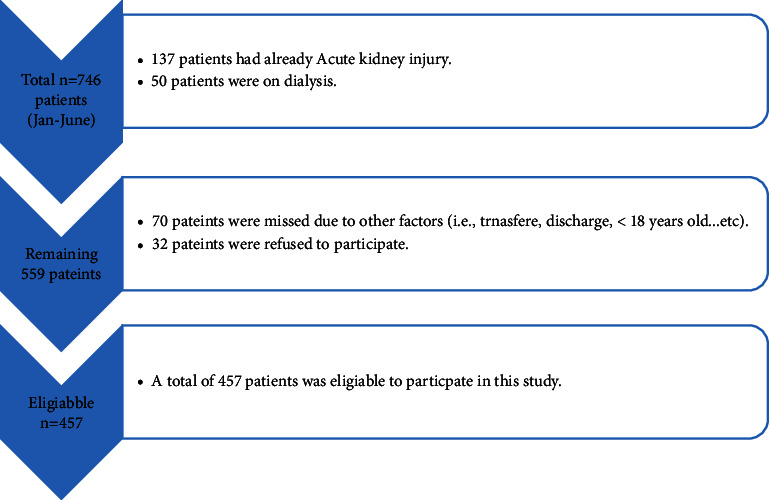
Eligible criteria for patients' participation.

Figure 1 illustrates the inclusion and exclusion criteria.

Figure 1 illustrates that the total count of admitted patients in the MICU at JUH amounted to 746 patients. Utilizing G*∗*Power software, it was determined that a minimum sample size of 254 participants was deemed sufficient. Out of these, 457 patients met the inclusion criteria, indicating a commendable response rate of 61.2%. It is worth noting that this number of participants surpasses the initially calculated sample size requirement. Consequently, this sample size was deemed robust and capable of providing a 95% confidence interval [16].

### 2.5. Data Collection and Definitions

At the time of admission to the MICU, we collected data on baseline characteristics such as demographic factors and comorbidities such as diabetes mellitus (DM), hypertension (HTN), cardiovascular diseases, chronic pulmonary disease, chronic liver diseases, chronic kidney disease (CKD), active malignancy, neurological disease, and endocrinological disease. Furthermore, data on all medication lists before MICU admission were collected, such as those for angiotensin receptor blockers (ARBs) or ACEIs, aspirin, diuretics, statins, proton pump inhibitors (PPI), and NSAIDs. Moreover, baseline vital signs were recorded at admission, including heart rate, systolic blood pressure, and diastolic blood pressure. The main causes of admission to MICU were identified as follows: (1) acute pulmonary diseases including any lung pathology that indicates admission to the ICU without major hemodynamical changes or signs of sepsis as an acute exacerbation of baseline lung disease as chronic pulmonary disease, asthma exacerbation, lung fibrosis, and so on, and the new onset of lung pathology as pneumonia without causing hemodynamical changes, (2) acute gastroenterology including medical causes of gastrointestinal bleeding, decompensated liver failure, acute liver failure, etc. Sepsis is defined as meeting the international sepsis definitions using surviving sepsis campaign [17], (3) acute neurological disease is defined as any neurological disease, such as ischemic or hemorrhagic stroke, or acute neurological illness including myasthenia gravis, Guillain-Barré syndrome, or other neuropathic diseases that requires ICU admission, (4) endocrinological diseases, including diabetic ketoacidosis, myxedema coma, Addisonian crisis, and hypercalcemia or hypocalcemia, and (5) acute cardiac causes, including acute decompensated heart failure associated with infections or cardiac arrhythmia associated with infections. Some patients reported illnesses that did not fit the above definitions. Baseline measurements of renal function were obtained through the assessment of serum creatinine levels and estimated glomerular filtration rate (eGFR), which was calculated using the chronic kidney disease epidemiology collaboration formula. Furthermore, patients with CKD and a GFR below 60 mL/min/1.75 m^2^ were also considered for inclusion in the study.

### 2.6. Statistical Analysis

All statistical analyses were conducted using SPSS, version 23. Before conducting analysis, we assessed the normality of data using the Shapiro–Wilk test. In the descriptive analysis of the demographic distribution, categorical variables were presented as percentages, while continuous variables were expressed as mean ± standard deviation. AKI was defined in accordance with the Kidney Disease: Improving Global Outcomes (KDIGO) criteria. Given the unavailability of urine output data for all patients, the diagnostic criterion for AKI relied on serum creatinine levels. Our proposed KDIGO criteria for diagnosing AKI included an abrupt (within 48 hours) decline in kidney function, an absolute increase in serum creatinine of 0.3 mg/dL or more (≥26.4 *μ*mol/L), or a percentage increase in serum creatinine of 50% or more (1.5-fold from baseline). For patients with stage 3 or higher CKD, a 1.5-fold increase from baseline was considered a diagnostic criterion [18]. Missing data were appropriately addressed through imputation techniques. We conducted imputation to ensure that the analysis and results are based on a complete dataset, minimizing the potential biases associated with missing information. Multicollinearity is assessed by calculating the variance inflation factor (VIF) to evaluate the interaction between variables.

To examine the potential predictor factors for AKI, including baseline characteristics, medications, and laboratory data, bivariate and multivariate logistic regression analyses were conducted. Additionally, to identify the most significant risk factor for mortality in MICU patients, a multivariate model was developed using predictors that exhibited a *p* value of 0.05 or less in the univariate models. The adjusted odds ratio (AOR) and its corresponding 95% confidence interval (CI) were then calculated for the multivariate model.

## 3. Results

### 3.1. Demographic Factors

The Shapiro–Wilk test (*W* = 0.59, *p*=0.16) indicated that our data did not significantly deviate from a normal distribution. We present the characteristics of the study sample in Table 1. The average mean age was 63.8 ± 18 years (range: 18–97 years). More than half of the participants (50.5%) were men. Acute pulmonary diseases were considered the most common indication for admission to the ICU, followed by sepsis, with a percentage of 34.4% and 24.3%, respectively. Out of several comorbidities, HTN was the most prevalent disease with a percentage of 64.5%, while the prevalence of DM and chronic pulmonary diseases was 52.3% and 25.4%, respectively. The mean serum creatinine level at time of admission was 1.1 ± 1.1 mg/dl, and that in the AKI group was 1.2 ± 1.4 compared to 0.9 ± 0.8 with a *p* value of 0.03. The average eGFR at the time of admission was 67.3 ± 23.6 mL/min/1.73 m^2^. Approximately 33.6% (140 of the patients) had CKD (GFR < 60 mL/min/1.75 m^2^). Besides, 75.2% of patients were using PPI medications, while 23.9%, 53.2%, and 15.1% of patients were using ACEIs/ARBs, diuretics, and NSAIDs, respectively.

**Figure 2:**
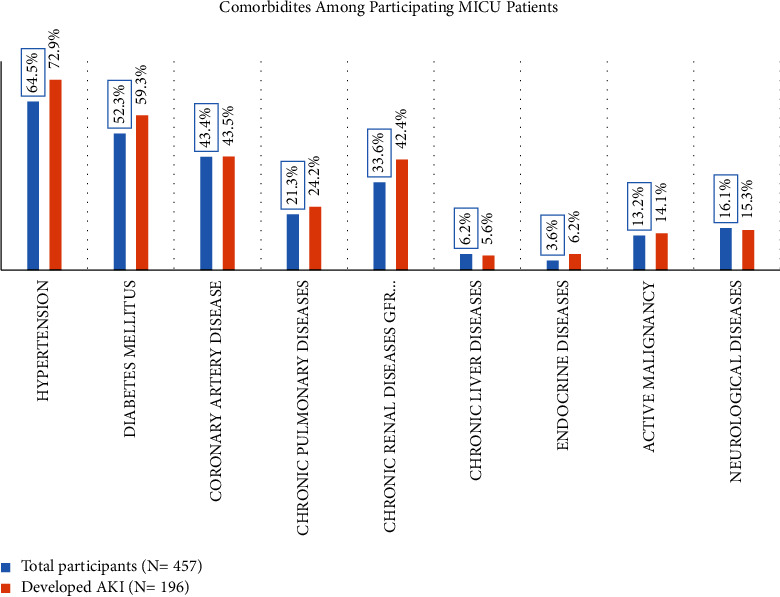
Comparison between comorbidities among total participants and those who developed AKI.

Among the 457 nonsurgical patients, a total of 196 patients (42.8%) developed AKI during their stay in the MICU. Notably, acute pulmonary diseases (*n* = 52, 34.5%) and sepsis (*n* = 40, 20.4%) were identified as the primary causes of admission in AKI patients. To provide further insights, we compared the percentages of total patients and the number of patients who developed AKI in relation to comorbidities. The findings are depicted in Figure 2.

### 3.2. Prevalence and Risk Factors of AKI

**Figure 3:**
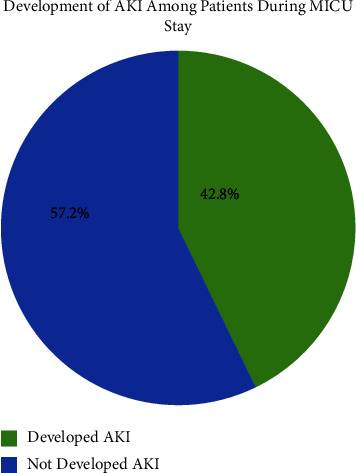
Development of AKI among patients during MICU stay (*N* = 457). AKI: acute kidney injury; MICU: medical intensive care unit.

The prevalence of AKI among MICU patients is shown in Figure 3.

A multivariate binary logistic regression model was utilized to assess significant variables associated with AKI, considering the findings from the univariate analysis (including baseline characteristics, comorbidities, medications at MICU admission, and laboratory results at admission) in Table 2. The analysis revealed that hypertension (AOR: 2.2; 95% CI: 1.1–4.6; *p*=0.03), chronic kidney disease (AOR: 2.1; 95% CI: 1.04–4.3; *p*=0.02), and NSAID use (AOR: 2.7; 95% CI: 1.3–5.9; *p*=0.01) were associated with an increased risk of AKI among nonsurgical patients. However, the use of ACE inhibitors/ARBs did not show an elevated risk of AKI (AOR: 1.04; 95% CI: 0.5–2.2; *p*=0.9). Additionally, serum urea level was found to be associated with AKI (AOR: 1.02; 95% CI: 1.014–1.02; *p*=0.001) (refer to Table 2 for detailed results). All VIF values were found to be within an acceptable range.

### 3.3. Interventions and Outcomes of AKI

The analysis illustrated a significant association between AKI and the use of vancomycin (*p* < 0.001), while the use of other antibiotics (colistin and aminoglycosides) did not show such an association (Table 3). Moreover, AKI was significantly associated with a higher mortality rate, with 35.7% in the AKI group compared to 26.9% in the non-AKI group (*p* < 0.001). Approximately 18.7% (34 patients) in the AKI group required renal replacement therapy. No significant association was found between the use of vasopressors and ventilators and AKI. Using a multivariate binary logistic regression model, vancomycin use was found to increase the risk of AKI among nonsurgical critically ill patients (AOR: 10; 95% CI: 4.7–24.0; *p* < 0.001).

### 3.4. Mortality in the MICU

The overall mortality rate for nonsurgical patients during their MICU stay was 26.9%. We determined the risk factors for mortality by univariate analysis, including age, baseline comorbidities, medications at admission, cause of admission, interventions during MICU stay such as ventilator and vasopressor use, antibiotics, AKI during MICU stay, and laboratory results. Moreover, we found that older age (AOR: 1.04; 95% CI: 1.04–1.06; *p*=0.003), use of diuretics (AOR: 2.12; 95% CI: 1.06–4.25; *p*=0.03), use of ventilators (2.19; 95% CI: 1.12–2.29; *p*=0.02), and use of vasopressors (AOR: 4.25; 95% CI: 2.1308.47; *p*=0.001) were significantly associated with the mortality rate. Statin use appears to be a protective factor for MICU patients (AOR: 0.42; 95% CI: 0.2–0.85; *p*=0.02). Surprisingly, using antibiotics such as vancomycin, fluoroquinolones, aminoglycosides, and colistin was not found to affect the mortality rate. Regarding outcome, it was revealed that nonsurgical patients with AKI during MICU stay had a higher mortality rate (AOR: 2.44; 95% CI: 1.07–5.56; *p*=0.03); however, we could not find a link between longer hospital stay and mortality rate (Table 4).

## 4. Discussion

This study reveals AKI prevalence and risk factors in critically nonsurgical patients admitted to JUH with medical conditions. We found that the prevalence of AKI is approximately 42.8%. Patients with AKI are at a higher risk of morbidity and mortality, have a longer hospital stay, and have an increased risk of developing CKD [1]. This may be related to the selected cutoff-point for the diagnostic criteria of developing AKI. A systematic review found that AKI among critically ill patients ranges from 14.4 to 39% [19]. Consistent with the findings of Melo et al. [20], in a recent systematic review encompassing studies from both developed and developing countries, the prevalence of AKI was also approximately 40%. This similarity in AKI prevalence further supports the existing body of literature across different regions. Another study, which published the results of a multination study, showed the overall AKI prevalence as 57.3% [21]. In different international multicenter studies, the occurrence rate of the condition in patients from both developing and developed countries was approximately 19% [22]. Results revealed that the mortality rate among all nonsurgical patients is 26.9% and significantly increases to 35.7% in patients who developed AKI during their MICU stay. Notably, this is considerably less than results reported in a previous study, which found the mortality rate to be 50–70% [23], and higher than the one in the previous study of Hashemian et al. [24], which reported a percentage of 19.7%. Other factors, such as the patient's condition, the severity of the illness, and older age, contribute to the mortality rate. Also, we found that comorbidities including DM, HTN, pulmonary diseases, and CKD are associated with a higher risk of developing AKI [25]. Previous studies showed an association between chronic pulmonary diseases and acute exacerbations of chronic obstructive pulmonary disease and the risk of AKI, which may be related to using positive pressure ventilators, developing hypoxemia and systemic inflammation, and different cytokines [26, 27]. This study concurs with a previous study by Cao et al. [27] and challenges that by Hamid et al. [28], which found that sepsis (74.3%) was the primary source of developing AKI among nonsurgical hospitalized critically ill patients in Malaysia.

PPIs are widely prescribed for gastrointestinal disorders that are predominant among older adults, including dyspepsia, gastric ulcer, and gastroesophageal reflux disease. Despite a high prevalence of PPI usage, we did not find an increased risk of AKI. This observation aligns with a study conducted by Lee et al. [29], where they also reported no significant association between preadmission PPI use and AKI in critically ill patients, even after accounting for demographic factors, illness severity, and the indication for PPI use.

We found that use of ACEI/ARB drugs is associated with AKI among nonsurgical patients. This study concurs with a previous study by Neyra and Leaf [30], which found that the patients who were taking ACEIs/ARBs at admission were at a higher risk of developing AKI. This can be explained by an increase in the mechanism of action of ACEI/ARB by decreasing intraglomerular pressure, which may contribute to AKI risk. Another study found that previous use of ACEI/ARB may be related to in-hospital mortality rate among MICU patients [31]. Long-term and continued use of ACEI/ARB after MICU discharge and during renal recovery is recommended, with cautious evaluation. It is widely known that NSAIDs directly interfere with renal function and increase the risk of AKI. We found that vancomycin is associated with a higher risk of AKI especially when exceeding the level of 20 mg/l. Other previous studies indicated that vancomycin drug increased AKI among ICU patients [8, 32].

Regarding mortality, we found that older age, diuretic drug use prior to hospital admission, and ventilator and vasopressor use during MICU stay are linked to an increased risk of death. Most antibiotic drugs used in critically ill patients are not associated with mortality rate, hospital length of stay, or malignancy. The risks of AKI and mortality rates were noted in a previous study [32]. Surprisingly, the use of diuretics is linked to a two-fold (2.12 times) increased mortality rate. In 2019, they found that diuretic drugs are useful tools in the management of AKI-associated volume overload, and they are improbable to be inherently toxic when used appropriately; however, they are also improbable to have a therapeutic role in AKI as a “stand-alone” therapy by preventing fluid overload [33]. Vasopressors are linked to an increased risk of death but did not cause AKI. This result requires more investigation because it may be related to early death even before developing AKI. Alternatively, this could be attributed to the clinical approach adopted in our institution, which involves the early initiation of vasopressors. This approach may result in reduced utilization of resuscitation fluids, decreased fluid accumulation, and potentially shorter durations of hypotension. These findings are consistent with those reported by Ospina-Tascón et al. [34], who demonstrated that the early initiation of vasopressors was not significantly associated with an increased risk of acute renal failure or the need for renal replacement therapy. A previous study found that inotrope use contributes to both AKI and mortality rates [35]. Invasive mechanical ventilator use is associated with a threefold increase in the odds of AKI in critically ill patients [36]. This may indicate that patients die early in the MICU, even before developing AKI.

Statin use was found to have a protective effect on critically ill patients. These findings align with a meta-analysis conducted by Yu et al. [37], which demonstrated that preadmission statin use was associated with favorable outcomes in critically ill patients, including lower short-term mortality rates.

### 4.1. Limitation

The utilization of a retrospective study conducted in a single-center hospital represents a limitation of this study. Insufficient data in medical records prevented the assessment of additional variables such as body mass index, urine output volume, and fluid management strategies, which also constitutes a potential limitation. We also did not include factors like nutritional status, genetics, and drug interactions in this study. Future research should explore these variables to enhance our understanding of AKI. Moreover, studying the quality of life among AKI patients is needed [38]. Nonetheless, this study benefited from a comprehensive evaluation of various preadmission and postadmission factors within the MICU, with minimal missing data points, highlighting the study's strengths. Moreover, this study is the first in the region to employ AKIN criteria for diagnosing AKI, adding novelty to the findings. However, considering the aforementioned limitations, a multicenter prospective study with a larger sample size is warranted to further explore these findings.

## 5. Conclusion

This study highlights that AKI is one of the most severe complications (42.8%) among nonsurgical patients during MICU stay in JUH and is associated with short- and long-term morbidity and mortality outcomes (35.7%). Identified risk factors for AKI are DM, HTN, CKD, pulmonary causes, smoking, and home medications, including ACEIs/ARBs and NSAIDs. Older patients using diuretics, vasopressors, and ventilators are at higher risk of mortality. The use of statins has a protective effect with a lower mortality rate. Further prospective studies are required to identify the risk factors for AKI in nonsurgical critically ill patients and provide certain preventive measures.

## Figures and Tables

**Table 1:** Baseline characteristics and factors contributing to the occurrence of AKI.

Baseline characteristics	Total sample *N* = 457	No AKI *N* = 261	Developed AKI during MICU stay *N* = 196	*p* value
Age group				0.72
M ± SD	63.8 ± 18.2	63.2 ± 17.6	64.2 ± 18.9	
18–60 years	181 (39.6%)	106 (58.6%)	75 (41.4%)	
>60 years	276 (60.4%)	155 (56.2%)	121 (43.8%)	
Gender				0.64
Female	226 (49.5%)	127 (56.2%)	99 (43.8%)	
Male	231 (50.5%)	134 (58.0%)	97 (42.0%)	
Smoking (yes %)	69 (16.5%)	47 (68.1%)	22 (31.9%)	**0.03** ^*∗*^
Comorbidities				
Hypertension (yes %)	269 (64.5%)	140 (52.0%)	129 (48.0%)	**0.002** ^*∗∗*^
Diabetes mellitus (yes %)	218 (52.3%)	113 (51.8%)	105 (48.2%)	**0.01** ^*∗∗*^
Coronary artery disease (yes %)	181 (43.4%)	104 (57.5%)	77 (42.5%)	0.91
Chronic pulmonary diseases (yes %)	110 (25.4%)	63 (57.3%)	47 (42.7%)	0.63
Chronic kidney diseases GFR <60 (yes %)	140 (33.6%)	65 (46.4%)	75 (53.6%)	**0.001** ^*∗*^
Chronic liver diseases (yes %)	26 (6.2%)	16 (61.5%)	10 (38.5%)	0.72
Endocrine (yes %)	13 (3.6%)	2 (15.4%)	11 (84.6%)	**0.002** ^*∗∗*^
Active malignancy (yes %)	55 (13.2%)	30 (54.6%)	25 (45.5%)	0.65
Neurological disease (yes %)	67 (16.1%)	40 (59.7%)	27 (40.3%)	0.76
Cause of admission				
Acute pulmonary diseases	157 (34.4%)	97 (61.2%)	60 (38.2%)	0.06
Acute gastroenterological diseases	73 (16.2%)	38 (52.0%)	35 (48.0%)	
Acute neurological diseases	54 (11.8%)	34 (63.0%)	20 (37.0%)	
Sepsis	111 (24.3%)	56 (50.4%)	55 (49.5%)	
Acute cardiovascular diseases	28 (6.2%)	15 (53.6%)	13 (46.4%)	
Endocrine disorders	12 (2.6%)	8 (66.7%)	4 (33.3%)	
Others	21 (4.6%)	10 (47.6%)	11 (52.4%)	
Medications				
ACEI or ARB (yes %)	104 (23.9%)	69 (66.4%)	35 (33.7%)	**0.03** ^*∗*^
NSAIDs (yes %)	66 (15.1%)	29 (43.9%)	37 (56.1%)	**0.02** ^*∗*^
Aspirin (yes %)	202 (46.3%)	106 (52.5%)	96 (47.5%)	0.07
Diuretics (yes %)	232 (53.2%)	134 (57.8%)	98 (42.2%)	0.83
Statin (yes %)	172 (39.4%)	94 (54.7%)	78 (45.4%)	0.43
PPI (yes %)	328 (75.2%)	188 (57.3%)	140 (42.7%)	0.91
Vital signs				
Systolic blood pressure (mmHg)	135.3 ± 34.6	135.7 ± 34.9	134.8 ± 34.3	0.90
Diastolic blood pressure (mmHg)	73.2 ± 23.4	75.5 ± 22.0	70.2 ± 24.4	**0.02** ^*∗*^
MAP (mmHg)	81.6 ± 38.6	83.5 ± 38.9	79.1 ± 38.3	0.24
Heart rate	91.8 ± 22.9	93.1 ± 23.9	90.15 ± 21.5	0.35
Respiratory rate	20.2 ± 3.1	20.2 ± 2.9	20.1 ± 3.3	0.91
Laboratory results				
Hemoglobin level	11.2 ± 6.0	11.7 ± 7.6	10.6 ± 2.6	**0.01** ^*∗∗*^
White blood count	11.6 ± 10.3	10.7 ± 7.0	12.65 ± 11.5	0.12
Platelets count	235.0 ± 124.2	239.7 ± 129.3	228.9 ± 117.2	0.53
Serum sodium level	139.1 ± 8.9	139.1 ± 10.0	139.2 ± 7.2	0.42
Serum glucose	170.9 ± 119.0	170.7 ± 126.0	171.1 ± 110.2	0.88
Serum urea	79.2 ± 65.0	54.9 ± 42.6	110.8 ± 74.8	**<0.001** ^*∗∗*^
Serum potassium	4.4 ± 0.9	4.2 ± 0.7	4.6 ± 1.1	**<0.001** ^*∗∗*^
Serum bicarbonate	22.4 ± 7.2	24.0 ± 6.4	20.6 ± 7.6	**<0.001** ^*∗∗*^
Serum PH	7.38 ± 0.1	7.4 ± 0.1	7.36 ± 0.1	**<0.001** ^*∗∗*^
Serum albumin	3.3 ± 0.9	3.4 ± 1.1	3.22 ± 0.7	0.05
Serum CRP	100.9 ± 132.2	95.2 ± 129.3	108.4 ± 135.8	0.46
Serum creatinine	1.1 + 1.1	0.9 + 0.8	1.2 + 1.4	**0.03** ^*∗*^
eGFR	67.3 ± 26.6	72.6 ± 23.4	60.2 ± 28.9	**<0.001** ^*∗∗*^

AKI: acute kidney injury; ICU: intensive care unit; ACEI: angiotensin-converting enzyme inhibitor; ARB: angiotensin receptor blocker; NSAIDs: nonsteroidal anti-inflammatory drugs; PPI: proton pump inhibitor; GFR: glomerular filtration rate; MAP: mean arterial pressure. ^*∗*^*p* value ≤0.05; ^*∗∗*^*p* value ≤0.01. The bold values are significant with one star <0.05, with two stars <0.001.

**Table 2:** Factors associated with AKI—bivariate and multivariate logistic regression analyses.

Variables	OR (95% CI)	VIF	*p* value
Smoking	0.7 (0.3–1.5)	1.04	0.41
Diastolic blood pressure	1.0 (0.9–1.0)	1.03	0.22
Comorbidities			
Diabetes	0.9 (0.5–1.9)	1.33	0.85
Hypertension	2.2 (1.1–4.6)	1.35	**0.03** ^*∗*^
Chronic kidney diseases with GFR <60	2.1 (1.04–4.3)	1.16	**0.02** ^*∗*^
Medications			
NSAIDs	2.7 (1.3–5.9)	1.10	**0.01** ^*∗∗*^
ACEIs/ARBs	1.04 (0.5–2.2)	1.17	0.91
Laboratory data			
Hemoglobin level	0.9 (0.8–1.03)	1.05	0.12
Urea	1.02 (1.01–1.02)	1.03	**0.001** ^*∗∗*^
PH	0.09 (0.004–2.0)	1.35	0.43
Serum potassium	1.2 (0.8–1.9)	1.38	0.41
HCO_3_	0.97 (0.92–1.02)	1.28	0.22

Included in the multiple logistic regression analysis; AKI: acute kidney injury; ACEIs: angiotensin-converting enzyme inhibitors; ARBs: angiotensin receptor blockers; CI: confidence interval; OR: odds ratio; VIF: variance inflation factor. ^*∗*^*p* value ≤0.05; ^*∗∗*^*p* value ≤0.01. The bold values are significant with one star <0.05, with two stars <0.001.

**Table 3:** MICU interventions and outcomes.

Interventions	All patients *N* = 457	No AKI	Developed AKI *n* = 196	*p* value
Noninvasive ventilation	171 (37.4%)	100 (58.5%)	71 (41.5%)	0.28
Ventilators (yes %)	179 (39.1%)	69 (50.4%)	65 (49.6%)	0.24
Vasopressors (yes %)	102 (27.1%)	54 (52.9%)	44 (47.1%)	0.72
Antibiotics				
Aminoglycosides	32 (8.2%)	18 (56.3%)	14 (43.7%)	0.91
Colistin	77 (20.5%)	47 (61.0%)	30 (39%)	0.26
Vancomycin	162 (35.4%)	52 (32.1%)	110 (67.9%)	**<0.001** ^*∗∗∗*^
Fluoroquinolones	234 (62.2%)	131 (56%)	103 (44%)	0.62
Clinical outcomes				
Hospital stay (days)	13.44 ± 12.2	12.7 ± 11.5	14.5 ± 14.7	0.53
MICU stay (days)	9.0 ± 9.4	9.3 + 9.5	8.5 ± 9.4	0.37
Mortality (%)	123 (26.9%)	53 (43.1%)	70 (56.9%)	**<0.001** ^*∗∗∗*^

MICU: medical intensive care unit. ^*∗∗∗*^*p* value <0.001. The bold values are significant with three stars <0.001.

**Table 4:** Multivariate binary logistic regression analysis of factors associated with MICU mortality (*N* = 457).

Variables	OR (95% CI)	*p* value
Baseline admission data		
Age	1.04 (1.04–1.06)	**0.003** ^*∗∗*^
Comorbidities: malignancy	1.38 (0.55–3.42)	0.49
Medications		
Diuretics	2.12 (1.06–4.25)	**0.03** ^*∗*^
Statin	0.42 (0.21–0.85)	**0.02** ^*∗*^
Antiplatelet	1.37 (0.68–2.75)	0.42
Interventions during MICU stay		
Ventilators	2.19 (1.12–4.29)	**0.02** ^*∗*^
Vasopressors	4.25 (2.13–8.47)	**<0.001** ^*∗∗∗*^
Antibiotics during MICU stay		
Aminoglycoside	2.67 (0.84–8.53)	0.09
Fluoroquinolones	1.46 (0.65–3.83)	0.46
Colistin	1.83 (0.59–5.62)	0.35
Vancomycin	1.42 (0.52–3.83)	0.51
Outcome		
Hospital length of stay	1.02 (0.99–1.05)	0.11
AKI during MICU stay	2.44 (1.07–5.56)	**0.03** ^*∗*^

AKI: acute kidney injury; MICU: medical intensive care unit; CI: confidence interval; OR: odds ratio. Predictive power of final model. C statistics: 53.7%. ^*∗*^*p* value ≤0.05; ^*∗∗*^*p* value ≤0.01; ^*∗∗∗*^*p* value ≤0.001. The bold values are significant with one star <0.05, with two stars <0.01, with three stars <0.001.

## Data Availability

The data underlying the results presented in the study are available on the following link: https://figshare.com/articles/dataset/AKI_sav/22656421.
